# Effect of Tai Chi Chuan in Breast Cancer Patients: A Systematic Review and Meta-Analysis

**DOI:** 10.3389/fonc.2020.00607

**Published:** 2020-04-23

**Authors:** Xiao-Chao Luo, Jie Liu, Jia Fu, Hai-Yan Yin, Li Shen, Mai-Lan Liu, Lei Lan, Jian Ying, Xiu-Lan Qiao, Chun-Zhi Tang, Yong Tang

**Affiliations:** ^1^Acupuncture and Tuina School, Chengdu University of Traditional Chinese Medicine, Chengdu, China; ^2^Sichuan Provincial People's Hospital, Chengdu, China; ^3^Medical & Nursing School, Chengdu University, Chengdu, China; ^4^The School of Acupuncture, Moxibustion & Tuina, Hunan University of Traditional Chinese Medicine, Changsha, China; ^5^Key Laboratory of Sichuan Province for Acupuncture & Chronobiology, Chengdu, China; ^6^Chongqing Traditional Chinese Medicine Hospital, Chongqing, China; ^7^School of Acupuncture & Rehabilitation Medicine, Guangzhou University of Traditional Chinese Medicine, Guangzhou, China

**Keywords:** Tai Chi Chuan, breast cancer, physical and psychological symptoms, quality of life, meta-analysis

## Abstract

**Background:** Tai Chi Chuan(TCC), as a mind-body exercise, may have a positive impact on physical function and psychological well-being in breast cancer patients. The latest systematic review and meta-analysis of TCC for breast cancer was made 4 years ago and some new clinical trials about it were published. We remade a systematic review and meta-analysis to evaluate the effect of TCC in breast cancer patients.

**Methods:** In this systematic review and meta-analysis, we searched MEDLINE (via PubMed), EMBASE (via embase.com), CENTRAL, CNKI, COVIP, Wanfang, Chaoxing, CiNii, J-SSTAGE, DBpia, and ThaiJO with no language restrictions from inception to December 31, 2018 (updated on February 16, 2020), for randomized clinical trials comparing TCC with non-exercised therapy in breast cancer patients. The primary outcome was quality of life in patients with breast cancer and data pooled by a random-effects model. Subgroup analyses were conducted to estimate the effect of different durations of TCC for breast cancer patients. This study was registered in PROSPERO, number CRD 4201810326.

**Results:** Fifteen articles involving a total of 885 breast cancer participants were included in this review. Compared with non-exercised therapy, TCC had a significant effect on quality of life in breast cancer patients (SMD = 0.37, 95% CI 0.15–0.59, *p* = 0.001), and subgroup analysis found that TCC showed beneficial effect in 12 weeks and 25 weeks (12 weeks: SMD = 0.40, 95% CI 0.19–0.62, *p* = 0.0003; 25 weeks: SMD = 0.38, 95% CI 0.15–0.62, *p* = 0.002). Meta-analyses of secondary outcomes showed that 3 weeks TCC increased shoulder function (SMD = 1.08, 95% CI 0.28–1.87, *p* = 0.008), 12 weeks TCC improved pain (SMD = 0.30, 95% CI 0.08–0.51, *p* = 0.007), shoulder function (SMD = 1.34, 95% CI 0.43–2.25, *p* = 0.004), strength of arm (SMD = 0.44, 95% CI 0.20–0.68, *p* = 0.0004), and anxiety (MD = −4.90, 95% CI −7.83 to −1.98, *p* = 0.001) in breast cancer patients compared with the control group.

**Conclusions:** TCC appears to be effective on some physical and psychological symptoms and improves the quality of life in patients with breast cancer. Additional randomized controlled trials with a rigorous methodology and low risk of bias are needed to provide more reliable evidence.

## Introduction

Although breast cancer is the most commonly diagnosed cancer and being the leading cause of cancer-related mortality in female (1–3), the long-term survival rates after a diagnosis of breast cancer are steadily rising in recent years (4–6). In the meantime, more patients with breast cancer are facing persistent symptoms and side-effects after diagnosis and treatment, such as fatigue (7–10), cognitive limitations (7, 9), depression (7, 9), anxiety (9), sleep problems (7, 9), and pain (9, 11). To address the persistent symptoms, complementary and alternative medicine (CAM), a non-mainstream medicine used together with or in place of conventional medicine, is recommended as supportive care strategies during and following the treatment (12).

Complementary therapies, especially mind-body practices, are effective approaches to manage breast cancer symptoms, and side-effects of treatment (12). For breast cancer patients, physical and psychosocial therapies improve physical function, and emotional disorders (13–15), and moreover, post-diagnostic physical activity reduces cancer-related mortality (16–18). Tai Chi Chuan (TCC) and Qigong are complementary therapies involving physical and psychological aspects (19). They are ancient Chinese mind-body exercises that both combine meditation, breathing, relaxation, and physical activity (20–22). Traditional TCC is usually a series of elaborate, lengthy, and complex movements, while Qigong is a simpler and more repetitive exercise (22, 23). TCC is a martial art that was gradually simplified and made into a common sport in 1950s (24). Nowadays, TCC as a sport focuses more on body environment and mind-body interaction (25). When TCC is performed for health and energy enhancement, it is a form of Qigong (26). Qigong produces more than a dozen forms to keep people healthy (22). Because other forms of Qigong are different from the way of practicing TCC, this review only focused on the effects of TCC on breast cancer patients.

TCC with slow, supple, graceful, curved, spiral and sequential motions, is a mild-to-moderate intensive whole-body exercise incorporating meditation into breathing control (20, 27, 28). TCC improves pain and anxiety in patients with fibromyalgia (29), exercise self-efficacy and mood disturbance in patients with chronic heart failure (30), depression and physical function in patients with osteoarthritis (31), and balance and tumble in patients with Parkinson's disease (32). TCC for more than 8 weeks reduces cancer-related fatigue, especially in breast or lung cancer patients (33, 34). In senior female cancer survivors, TCC affects systolic blood pressure and cortisol area-under-curve which may regulate the endocrine system (35). In general, TCC plays a good role in improving the quality of life (QOL) in cancer patients (36). TCC is recommended to patients with chronic conditions for multi-effects, easily learning, good safety, and low-cost (28).

Breast cancer is a chronical clinical setting with persistent symptoms of body and mind, and TCC may have a positive effect on it. Several clinical trials found TCC reduced inflammatory responses and improved quality of life, muscle strength, shoulder function, bone formation, and insomnia in breast cancer patients (37–41). Two early reviews published in 2007 and 2010, respectively, both found that the effect of TCC for breast cancer patients to improve QOL and symptoms was not definite (42, 43). In a systematic review and meta-analysis published in 2015, Pan and colleagues considered TCC had a significant effect on improving handgrip strength and limb elbow flexion, but failed to improve QOL, physical and emotional well-being, pain, and body mass index (44).

Previous reviews pooled studies ignoring the influence of diverse exercise durations and control therapies, and found limited evidence that TCC was beneficial on physical and psychosocial capacity in breast cancer patients. Additionally, some new clinical trials explored TCC for breast cancer have been published. Therefore, a systematic review and meta-analysis including the latest randomized clinical trials of TCC for breast cancer is necessary to be done. This systematic review and meta-analysis are performed to evaluate the effect of TCC on QOL and psychosomatic outcomes in breast cancer patients.

## Methods

### Search Strategy

The report of this systematic review and meta-analysis is followed by the Preferred Reporting Items for Systematic Reviews and Meta-analyses (PRISMA) (45). The checklist of PRISMA is in Supplement Table 1. We searched English databases (MEDLINE, EMBASE, and CENTRAL), Chinese databases (CNKI, CQVIP, Wanfang, Chaoxing), Japanese databases (CiNii, and J-SSTAGE), Korean database (DBpia), and Thai database (ThaiJO) from inception to December 31, 2018 (updated on February 16, 2020). MEDLINE and EMBASE were available for consultation through PubMed and embase.com, respectively. The following search terms in various relevant combinations were used to screen potential studies: tai^*^ji^*^, tai^*^chi^*^, breast cancer, breast tumor, breast neoplasm, breast carcinoma. Example of search strategy is in Supplement Table 2. Language restrictions were not part of data searches. The reference lists of identified original or review studies were searched manually for further articles.

### Selection Criteria

The following inclusive selection criteria were applied: (I) participants were adult female patients who were diagnosed breast cancer through pathology with any tumor stage; (II) intervention measure was TCC, such as Yang-style TCC, Chen-style TCC, Wu-style TCC, Sun-style TCC, 24 simplified TCC, or movements of TCC; (III) compared intervention was non-exercised treatment, such as standard support therapy (a psychotherapy for education and peer discussion on nutrition, exercise, stress, cancer risk, and fatigue) (46, 47), usual health care (regular check-ups, medication, and health education by health care workers), or blank control; (IV) primary outcome was QOL and the measurement was not limited [e.g., the Medical Outcomes Study 36-Item Short-Form Health Survey (MOS SF-36), the World Health Organization Quality of Life Brief Questionnaire (WHOQOL-BREF), the Functional Assessment of Chronic Illness Therapy-Fatigue (FACIT–F), the Functional Assessment of Cancer Therapy–Breast (FACT-B), and the Generic Quality of life Inventory 74 (GQOLL 74)]; secondary outcomes were pain, shoulder function, strength of arm, anxiety, and other clinical outcomes; (V) only randomized controlled trial (RCT) was included. Exclusive selection criteria: (I) participants were not only breast cancer patients; (II) experimental intervention was TCC combining other exercise; (III) control intervention included other exercise methods, such as yoga, physical activity, aerobics; (IV) data of outcomes couldn't be acquired; (V) non-randomized clinical trial.

### Data Extraction and Quality Assessment

Information extracted from each eligible study through electronic form containing the first author, year of publication, country of origin, number of participants, age, status of cancer, current treatment, experimental intervention, duration and frequency of TCC, controlled intervention, and outcomes. Data from eligible studies were extracted independently by two investigators (XL, LS). The risk of bias was assessed by the Cochrane Collaboration's tool with seven items: random sequence generation, allocation concealment, blinding of participants and personnel, blinding of outcome assessment, incomplete outcome data, selective reporting, and other bias (48). We used the Grades of Recommendations Assessment, Development and Evaluation (GRADE) system to evaluate the certainty of outcomes (49). Any disagreements were resolved by discussion and consensus.

### Data Analysis

We tried to make meta-analysis when the number of eligible studies was more than one in each outcome and data were combined by Review Manager (version 5.3, Cochrane Library). We selected a random-effect model to pool data (50–53). Mean difference (MD)/standardized mean difference (SMD) and 95% confidence interval (CI) were calculated. If outcome with the same measurement method, we selected MD, otherwise chosen SMD. Heterogeneity across studies was tested by the *I*^2^ statistic. *I*^2^ is regarded of 25, 50, and 75% as low, moderate, and high amount of heterogeneity, respectively (54). In the Cochrane Handbook, more than 50% of *I*^2^ are regarded as may material heterogeneity (55). We did subgroup analysis based on different TCC durations. A two-tailed *p* ≤ 0.05 was considered as a criterion for statistical significance.

### Registration

This study was registered in PROSPERO, number CRD 4201810326.

## Results

### Study Selection and Characteristics

**Figure 1:**
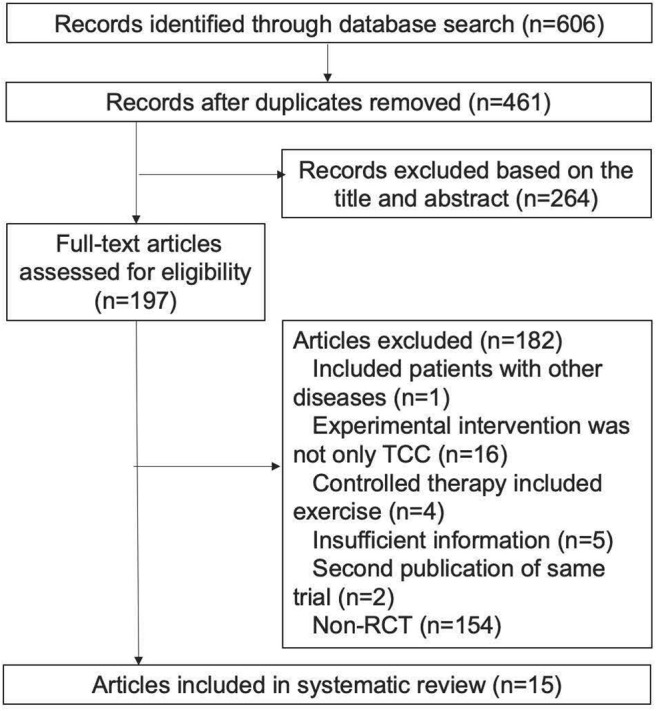
Literature search and screening process.

By searching English, Chinese, Japanese, Korean, and Thai databases, we obtained 606 records, of which 409 records were excluded for irrelevance, or duplication through the titles and abstracts. The full-text of the remaining 197 articles were retrieved for more detailed evaluation, and 182 articles were excluded (Figure 1). Fifteen articles (40, 41, 56–68) met the inclusion criteria containing 885 participants (447 in the TCC group, 438 in the control group). Six articles (40, 41, 65–68) came from the USA, eight (56–61, 63, 64) from China and one (62) from Thailand. The types of TCC were 15-move short-form of Yang-style TCC (40, 65–68), Chen-style TCC (58, 60), Tai Chi Yunshou (63), 18-form TCC (62), 24-form TCC (57, 59, 61, 64), 8-form TCC (56), and the other article (41) didn't mention the specific form of TCC. Duration of TCC was from 12 weeks to 6 months. The frequency of TCC was 120 min per week (41), 40–60 min per session and three sessions a week (40, 61, 62, 65–68), 20–30 min per session and two sessions per day (57–60, 63, 64), and at least 40 min per session for twice a day (56). TCC was performed during chemotherapy or after conventional therapy. Some trials (56–58, 61, 63, 64) used TCC intervention after surgery to alleviate the side effects of operation. The interventions of control groups were cognitive behavioral therapy (41), standard or psychosocial support therapy (40, 65–68), usual care (56, 62), and routine rehabilitation training (57–61, 63, 64). The interest control treatments had cognitive behavioral therapy (41), standard or psychosocial support therapy (40, 65–68), and blank control (56–64). The cognitive behavioral therapy involves re-establishing a consistent sleep-wake schedule, sleep restriction, relaxation, sleep hygiene education and cognitive procedures (69). The extracted information of eligible studies is in Table 1.

**Table 1:** Characteristics of the eligible articles.

**Study**	**Country**	**Patients (*****N*****)**		**Mean age(y)**		**Status**	**Treatment condition**	**TCC intervention**	**Controlled intervention**	**Frequency Duration**	**Outcomes**
		**TCC**	**CON**	**TCC**	**CON**						
Han et al. (56)	China	23	21	46.39	45.52	I-III	After modified radical mastectomy and chemotherapy	Usual care+8-form TCC	Usual care	At least 40 min/session and 2 sessions /day, 5 days/week, 12 weeks	1. Fatigue; 2. Adverse event
Irwin et al. (41)	USA	45	45	59·6	60·0	–	Treatment at least 6 months before enrollment	Tai Chi Chih	Cognitive behavioral therapy	120 min/session, 3 sessions/week, 3 months	1. Insomnia treatment response; 2. Insomnia remission; 3. Sleep quality; 4. Sleep continuity
Wang et al. (57)	China	45	41	50·5		I-III	10 days after modified radical mastectomy	RRT + 24-form TCC	RRT	20 min/session, every morning and evening, 6 months	1. QOL(WHOQOLBREF); 2. Fatigue; 3. Sleep quality; 4. Anxiety; 5. Depression
Wang et al. (58)	China	44	46	53·64	51·74	–	10 days after modified radical mastectomy	RRT + Chen-style TCC	RRT	20 min/session, every morning and evening, 3 months	1. QOL(FACT-B); 2. Neer shoulder function score
Zhu et al. (59)	China	47	48	45·51		–	After modified radical mastectomy	RRT +24-form simplified TCC	RRT	20 min/session, every morning and evening, 3 months	1. Pain; 2. Activities of daily living; 3. Range of motion; 4. Strength of arm
Wang et al. (60)	China	75	74	51·4	50·58	–	10 days after modified radical mastectomy	RRT + Chen-style TCC	RRT	20 min/session, every morning and evening, 6 months	Self-rating anxiety scale
Lv et al. (61)	China	50	49	48·61		–	After modified radical mastectomy andchemotherapy	RRT + 24-form simplified TCC	RRT	90 min/session, 3 sessions/week, 6 months	1. QOL(MOSSF-36); 2. Pain; 3. Activities of daily living; 4. Range of motion; 5. Strength of arm
Thongteratham et al. (62)	Thailand	15	15	–		0-IIIb	Completion of treatment at least 1 year before enrollment	Usual care + 18-form TCC	Usual care	60 min/session, 3 sessions/week, 12 weeks	1. QOL(FACT-B); 2. Rosenberg Self-esteem; 3. Fatigue Symptom Inventory; 4. Cortisol
Li et al. (63)	China	29	28	47·56		0-IIIb	7 days after modified radical mastectomy	RRT + Tai Chi Yunshou	RRT	30 min/session, at 7:00 and 17:00, 6 months	1. QOL(WHOQOLBREF); 2. Edema of upper extremity; 3. Function of shoulder joint; 4. Muscle strength
Wang et al. (64)	China	63	71	47·19		I-III	10 days after modified radical mastectomy	RRT + 24-form simplified TCC	RRT	20 min/session, every morning and evening, 180 days	1. QOL(WHOQOLBREF); 2. Pain; 3. Activities of daily living; 4. Range of motion; 5. Strength of arm
Sprod et al. (65)	USA	11	10	54·33	52·70	0–IIIb	Chemotherapy, radiotherapy, hormone therapy, or none	A 15-move short-form of Yang-style TCC	Standard support therapy	60 min/session, 3 sessions/week, 12 weeks	1. QOL(MOSSF-36); 2. Pain; 3. Biomarkers (IL-6, IL-8, IGF-1, IGFBP-1, IGFBP-3, glucose, insulin, cortisol)
Janelsins et al. (66)	USA	9	10	54·33	52·70	0–IIIb	Chemotherapy, radiotherapy, hormone therapy, or none	A 15-move short-form of Yang-style TCC	Psychosocial support therapy	60 min/session, 3 sessions/week, 12 weeks	1. Biomarkers (insulin, IGF-1, IGFBP-1, IGFBP-3, IL-6, IL-2, IFN-γ); 2. BMI; 3. Body composition
Peppone et al. (40)	USA	7	9	53·8	52·6	0–IIIb	Treatment completed from 1 to 30 months before enrollment	A 15-move short-form of Yang-style TCC	Standard support therapy	60 min/session, 3 sessions/week, 12 weeks	1. Bone-specific alkaline phosphatase; 2. N-telopeptides of type I collagen; 3. Bone Remodeling Index; 4. Biomarkers (IGFBP-1, IGFBP-3, cortisol, IL-2, IL-6, IL-8)
Mustian et al. (67)	USA	11	10	52		0–IIIb	Chemotherapy, radiotherapy, hormone therapy, or none	A 15-move short-form of Yang-style TCC	Psychosocial support therapy	60 min/session, 3 sessions/week, 12 weeks	1. QOL(FACIT-F); 2. Strength; 3. Flexibility; 4. Aerobic capacity
Mustian et al. (68)	USA	11	10	52		0–IIIb	Chemotherapy, radiotherapy, hormone therapy, or none	A 15-move short-form of Yang-style TCC	Psychosocial support therapy	60 min/session, 3 sessions/week, 12 weeks	1. Aerobic capacity; 2. Strength; 3. Flexibility; 4. Weight; 5. BMI;6. Body composition

*TCC, Tai Chi Chuan; CON, control; SD, standard deviation; QOL, quality of life; MOSSF-36, Medical Outcomes Study 36-Item Short-Form Health Survey; IL-6, interleukin-6; IL-8, interleukin-8; IGF-1, insulin-like growth factor-1; IGFBP-1, insulin-like growth factor-binding protein-1; IGFBP-3, insulin-like growth factor-binding protein-3; IL-2, interleukin-2; IFN-γ, interferon- γ; BMI, body mass index; FACIT-F, Functional Assessment of Chronic Illness Therapy-Fatigue; RRT, routine rehabilitation training; WHOQOLBREF, World Health Organization Quality of Life Brief Questionnaire; FACT-B, Functional Assessment of Cancer Therapy–Breast*.

### Risk of Bias

**Figure 2:**
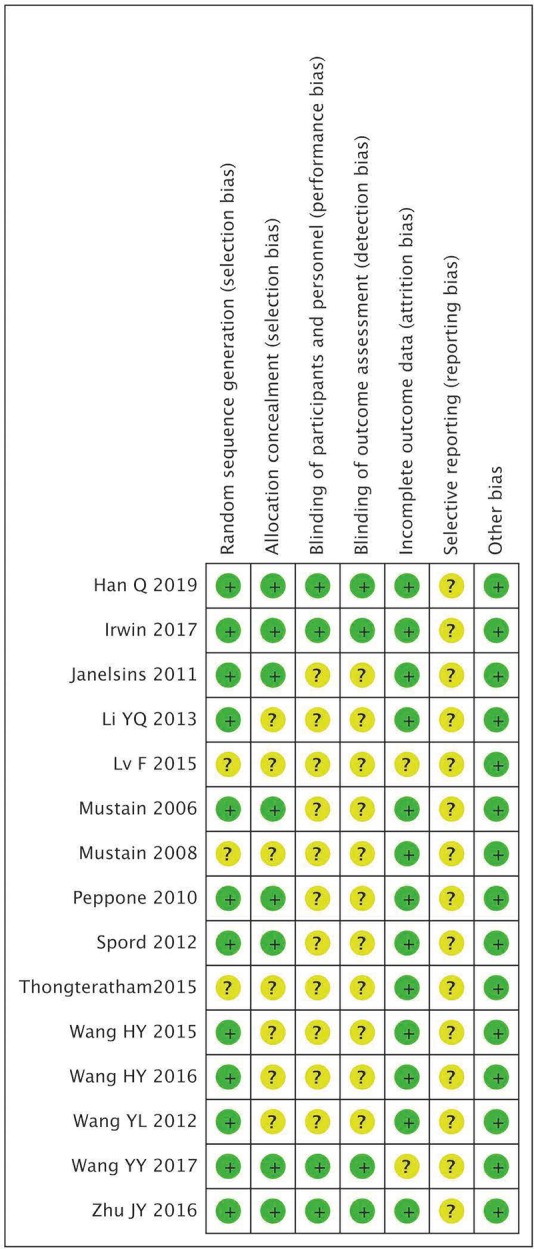
Risk of bias graph summary.

According to the Cochrane Collaboration's tool, risk of bias analysis was assessed to evaluate the quality of included studies. For eligible articles, 80% had low risk of random sequence generation and 54% had low risk of allocation concealment in selection bias; 73% had unclear risk of blinding both in performance and detection bias; 87% had low risk of incomplete outcome data in attrition bias; reporting bias was unclear because the proposals of these trials were not available (Figure 2). Publication bias was not analyzed because the number of eligible studies in each meta-analysis was <10 and conceivable publication bias existed in each meta-analysis.

### Meta-Analysis of TCC for QOL in Breast Cancer Patients

**Figure 3:**
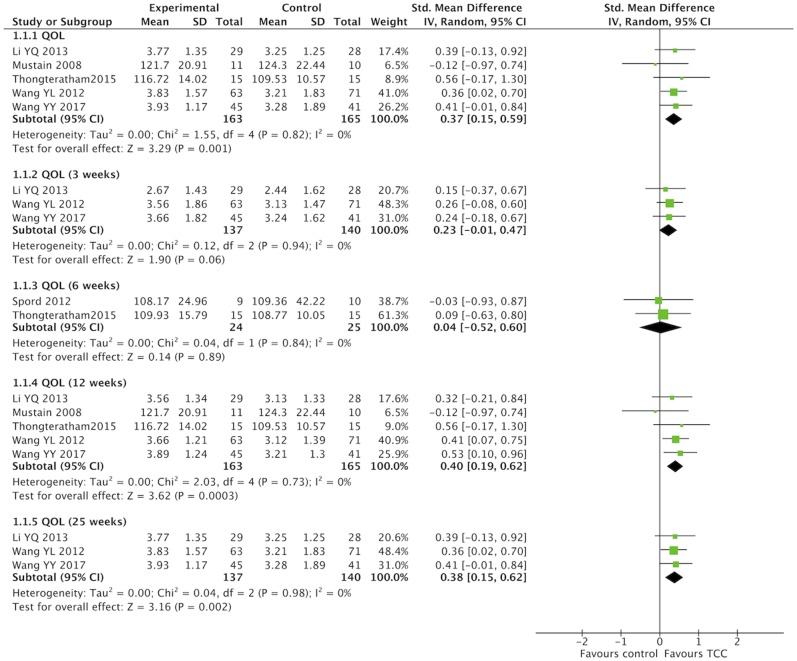
Meta-analyses of Tai Chi Chun for quality of life in breast cancer patients compared with non-exercised therapy. Standardized mean difference and 95% confidence interval are calculated.

QOL were evaluated with different scales in studies that performed a meta-analysis. One study (65) used the MOS SF-36 to assess general QOL, and three studies (57, 63, 64) chosen the WHOQOL-BREF. One study (67) used the FACIT–F to assess QOL in chronic disease, while another study (62) measured cancer-special QOL with the FACT-B. In this meta-analysis, SMD were calculated for different measures of QOL. TCC had a positive effect on QOL in breast cancer patients compared with the non-exercised therapy (SMD = 0.37, 95% CI 0.15–0.59, *p* = 0.001, *I*^2^ = 0%) (Figure 3). Subgroup meta-analyses of 12 and 25 weeks durations showed that TCC improved QOL in breast cancer patients (12 weeks: SMD = 0.40, 95% CI 0.19–0.62, *p* = 0.0003, *I*^2^ = 0%; 25 weeks: SMD = 0.38, 95% CI 0.15–0.62, *p* = 0.002, *I*^2^ = 0%), but meta-analyses of 3 and 6 weeks both were no statistical significance (3 weeks: SMD = 0.23, 95% CI −0.01 to 0.47, *p* = 0.06, *I*^2^ = 0%; 6 weeks: SMD = 0.04, 95% CI −0.52 to 0.60, *p* = 0.89, *I*^2^ = 0%).

### Meta-Analysis of TCC for Pain, Shoulder Function, Strength of Arm, Anxiety, and Fatigue in Breast Cancer Patients

**Figure 4:**
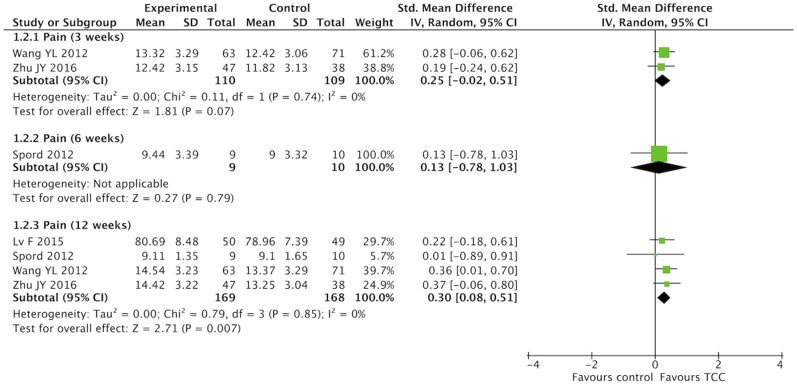
Meta-analyses of Tai Chi Chun for pain in breast cancer patients compared with non-exercised therapy. Standardized mean difference and 95% confidence interval are calculated.

**Figure 5:**
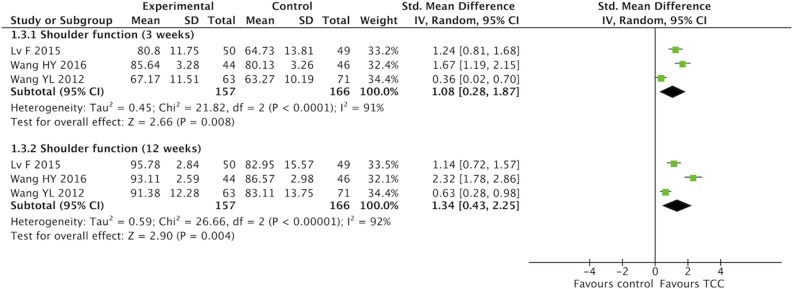
Meta-analyses of Tai Chi Chun for shoulder function in breast cancer patients compared with non-exercised therapy. Standardized mean difference and 95% confidence interval are calculated.

**Figure 6:**
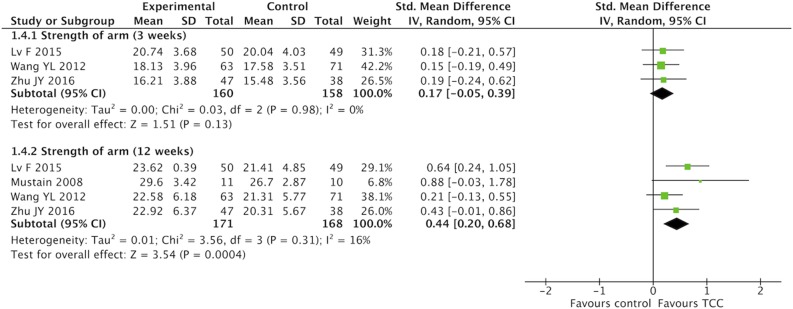
Meta-analyses of Tai Chi Chun for strength of arm in breast cancer patients compared with non-exercised therapy. Standardized mean difference and 95% confidence interval are calculated.

**Figure 7:**
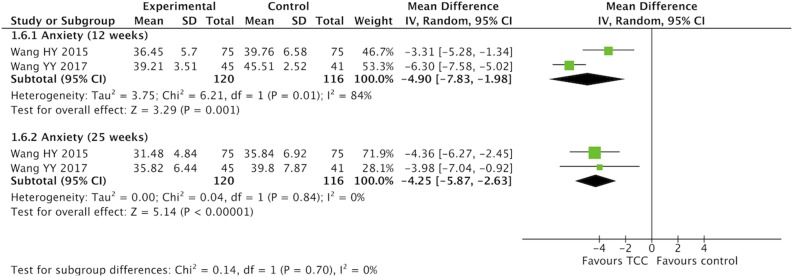
Meta-analyses of Tai Chi Chun for anxiety in breast cancer patients compared with non-exercised therapy. Mean difference and 95% confidence interval are calculated.

**Figure 8:**
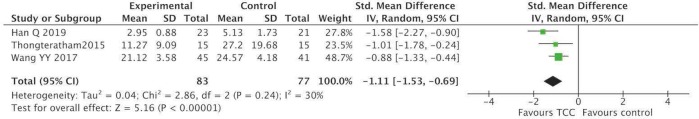
Meta-analyses of Tai Chi Chun for fatigue in breast cancer patients compared with non-exercised therapy. Standardized mean difference and 95% confidence interval are calculated.

For secondary outcomes, we did meta-analyses of pain, shoulder function, strength of arm, anxiety, and fatigue, respectively. The heterogeneity was high among the eligible studies in meta-analysis of TCC for shoulder function, even though we did sensitive analysis trying to delete one of the three included studies successively. In the meta-analyses of shoulder function and anxiety, the included studies were placed on one side of the no effect line, respectively. Therefore, we did meta-analyses of shoulder function and anxiety overlooking the heterogeneity. Meta-analyses showed that TCC was beneficial to alleviating pain (SMD = 0.30, 95% CI 0.08–0.51, *p* = 0.007, *I*^2^ = 0%) (Figure 4), recovering shoulder function (SMD = 1.34, 95% CI 0.43–2.25, *p* = 0.004, *I*^2^ = 92%) (Figure 5), increasing strength of arm (SMD = 0.44, 95% CI 0.20–0.68, *p* = 0.0004, *I*^2^ = 16%) (Figure 6), easing anxiety (MD = −4.25, 95% CI −5.87 to −2.63, *p* < 0.00001, *I*^2^ = 80%) (Figure 7), and relieving fatigue (SMD = −1.11, 95% CI −1.53 to −0.69, *p* < 0.00001, *I*^2^ = 30%) (Figure 8) compared with non-exercised therapy in breast cancer patients. TCC increased shoulder function in 3 weeks duration, but improved pain, strength of arm, and anxiety in 12 weeks in breast cancer patients.

### GRADE Evidence of Outcomes

GRADE system was used to assess the evidence quality of outcomes in this review. Risk of bias might exist because method of randomization, allocation concealment, and blinding were not definite. Outcomes measured by different methods might produce inconsistency. Although the heterogeneity in shoulder function and anxiety outcomes was high, inconsistency might not be downgraded because all the eligible studies in meta-analyses were on one side of the no effect line. The eligible studies and participants of each meta-analysis were small that imprecision and publication bias might exist. In general, the certainty of the six outcomes was very low (Table 2).

**Table 2:** GRADE evidence profile of outcomes.

**Certainty assessment**								**No. of patients**		**Effect**	**Certainty**
**Outcomes**	**No. of studies**	**Study design**	**Risk of bias**	**Inconsis-tency**	**Indirect-ness**	**Impre-cision**	**Other considera-tions**	**TCC**	**CON**	**Absolute (95% CI)**	
QOL	5	RT	Serious^*^	Serious^†^	Not serious	Serious^‡^	Publication bias strongly suspected^§^	163	165	SMD 0·37 higher (0·15 higher to 0·59 higher)	⊕○○○○ VERY LOW
Pain	4	RT	Serious^¶^	Not serious	Not serious	Serious^‡^	Publication bias strongly suspected^§^	169	168	SMD 0·3 higher (0·08 higher to 0·51 higher)	⊕○○○○ VERY LOW
Shoulder function	3	RT	Serious ^||^	Not serious	Not serious	Serious^‡^	Publication bias strongly suspected^§^	157	166	SMD 1·34 higher (0·43 higher to 2·25 higher)	⊕○○○○ VERY LOW
Strength of arm	4	RT	Serious^**^	Serious^†^	Not serious	Serious^‡^	Publication bias strongly suspected^§^	171	168	SMD 0·44 higher (0·20 higher to 0·68 higher)	⊕○○○○ VERY LOW
Fatigue	3	RT	Serious^††^	Serious	Not serious	Serious^‡^	Publication bias strongly suspected^§^	83	77	SMD 1.11 lower (1.53 lower to 0.69 lower)	⊕○○○○ VERY LOW
Anxiety	2	RT	Serious^‡‡^	Not serious	Not serious	Serious^‡^	Publication bias strongly suspected^§^	120	116	MD 4·25 lower (5·87 lower to 2·63 lower)	⊕○○○○ VERY LOW

*TCC, Tai Chi Chuan; CON, control; CI, confidence interval; QOL, quality of life; RT, randomized trials; SMD, standardized mean difference; MD, mean difference*.

* *2/5 studies didn't mention the method of randomization, 4/5 studies didn't mention allocation concealment and blinding*.

† *Measured by different scales*.

‡ *The sample size was small*.

§ *Number of eligible studies was small*.

¶ *1/4 studies didn't mention the method of randomization, 2/4 studies didn't mention allocation concealment, 3/4 studies didn't mention blinding*.

|| *1/3 studies didn't mention the method of randomization, 2/3 studies didn't mention allocation concealment and blinding*.

** *2/4 study didn't mention the method of randomization, 2/4 studies didn't mention allocation concealment and blinding*.

†† *1/3 study didn't mention the method of randomization*.

‡‡ *1/2 study didn't mention allocation concealment and blinding*.

## Discussion

This systematic review and meta-analysis concentrated on the QOL and psychosomatic symptoms in women breast cancer patients comparing TCC with non-exercise therapy.

Compared with the prior reviews (43, 44), this study searched more different language databases, included more RCTs, and made subgroup analysis on the basis of TCC duration. Lee et al. (43) and Pan et al. (44) made a systematic review of TCC for breast cancer in 2010 and 2015, respectively. Lee retrieved English, Chinese, and Korean databases, and Pan searched four English databases neglecting the different types of control intervention. In this review, we searched English, Chinese, Japanese, Korean, and Thai databases setting non-exercised therapy as the control treatment. Lee's review included seven studies, of which three were RCTs and four were non-RCTs, and only one meta-analysis of two RCTs was conducted; nine RCTs met the inclusion criteria for the Pan's review, and two to six studies were included in the meta-analyses; 15 RCTs were eligible in this review, but no more than five RCTs included in each meta-analysis. This review included 10 new RCTs (41, 56–64) which did not presented by Pan et al. (44) and might affect the results of meta-analysis. Pan did not search Chinese databases, while we searched four Chinese databases and found eight new Chinese articles (56–61, 63, 64) that met the inclusion criteria. The other two new studies (41, 62) were published in English from Thai and English databases, respectively. We made subgroup analysis according to the exercise time of TCC, and found that 12 weeks TCC was more effective than 3 or 6 weeks TCC in improving QOL, pain, and strength of arm in breast cancer patients. However, the two previous reviews did not conduct subgroup analysis according to the characteristics of TCC.

The findings of these two published reviews are quite different from those of our study. Lee et al. (43) found that TCC had no significant effect on QOL, physical outcomes (fatigue, body mass index, heart rate, and blood pressure) and psychological variables (self-esteem and depression) in patients with breast cancer. Pan et al. (44) discovered that TCC had a positive effect on handgrip dynamometer strength and limb elbow flexion, but had no significant difference in general health-related QOL, pain, and body mass index in breast cancer patients. In this review, TCC had an improvement on QOL in breast cancer patients. In the meta-analysis of TCC for QOL, Lee's review included two small sample trials, and Pan's review involved studies reporting the same trials, while we pooled data from five RCTs. Pan's review discovered that TCC failed to alleviate pain in breast cancer patients, whereas TCC had an inverse result in this systematic review. The meta-analysis of pain in this review included some new studies which influenced the result. Meta-analyses of fatigue and anxiety in this review were changed between the previous two reviews. Although this study discovered that TCC could reduce fatigue and anxiety in breast cancer patients compared with the non-exercised group, the result is uncertain because of a small number of eligible studies and high risk of selection bias, performance bias, detection bias, and publication bias. TCC was associated with positive effects on handgrip dynamometer strength and limb elbow flexion in Pan's review, and in this review also revealed that TCC improved strength of arm and shoulder function including range of motion.

QOL, a common gauge of cancer treatment effect, is widely used to measure the health of breast cancer patients (70) QOL is a multidimensional assessment with physical (including function/disability and symptoms/complications concepts), mental (including emotional distress, psychological well-being, perceived cognitive functioning, and spiritual/existential concerns concepts) and social (including socioeconomic challenges, role/relationship changes, perceived support/satisfaction, and social participation concepts) domains (70). Yan's and Tao's systematic reviews found TCC failed to improve QOL in breast cancer survivors (71, 72), but this study showed 12 weeks TCC had a positive influence on QOL. Three studies in Yan's systematic review were not included in this review because the intervention therapy of two studies (73, 74) was Tai Chi combining aerobics, and another study (75) was a prospective longitudinal study from a larger randomized controlled trial. We included some new studies that might produce the different pooled result. TCC improved the physical and mental health domains of QOL in cancer survivors at a low-level evidence in a recent review (76). Due to the limited number of included studies in the meta-analysis of QOL, we conducted a meta-analysis of overall QOL total score rather than a meta-analysis of each QOL domain in breast cancer patients, but obtained a very low-level evidence. Heather Greenlee and colleagues (12) recommends Qigong as C-graded therapy to improve QOL in breast cancer patients. TCC is one kind of Qigong and can be considered as a complementary health approach for QOL in breast cancer patients.

In this review, TCC also improved pain, shoulder function, strength of arm, anxiety, and fatigue in breast cancer patients. Pain is one of the most common symptoms in cancer patients and can be caused by tumors, surgery, chemotherapy, radiation therapy, targeted therapy, supportive care therapies, and diagnostic procedures (77). Half of breast cancer patients have mild pain and 16% have moderate to severe pain in 1 year after surgery (78). Pain has a psychological effect making patients product anxiety, nervousness, and anger (79). Control of surgery-related and non-operative pain in patients with breast cancer is essential. In addition to pain, side-effects of surgery include depressed shoulder function and arm strength (80). Decreased arm muscle strength in breast cancer patients after chemotherapy is also measured (81). Breast cancer patients have suffered psychological disorders as well as physical symptoms. About one-third of breast cancer survivors feel fatigue (82), and 40% patients are anxious in the year after diagnosis (83). Fatigue correlates with depression and sleep disturbance in breast cancer patients (84). Breast cancer patients experience persistent physical and mental disorders which influence QOL after or during cancer diagnosis and treatment. Three months exercise reduces fatigue, anxiety, and depression in breast cancer patients (85). American Society of Clinical Oncology Breast Cancer Survivorship Care Guideline recommends that primary care clinicians should counsel breast cancer patients to engage in regular physical activity to reduce cancer-related fatigue, musculoskeletal symptoms, pain, and obesity (86). Previous meta-analyses discovered that TCC improved psychological well-being including reduced stress, anxiety, depression and mood disturbance, and physical function in people with cancer (36, 87). In this review, TCC improved pain, shoulder function, strength of arm, and anxiety in breast cancer patients in 12 weeks, and also increased QOL in 12 and 25 weeks. We recommend breast cancer patients for more than 12 weeks TCC to manage symptoms and enhance QOL finally.

TCC is not only active in breast cancer, but also in other cancers. TCC improves vigor in patients with lung cancer undergoing chemotherapy (88). In addition, TCC can alleviate fatigue, enhance neck and shoulder joints mobility, and improves sleep in nasopharyngeal carcinoma patients (89, 90). For cancer survivors undergoing chemotherapy, TCC improves cancer-related fatigue, self-efficacy, and QOL (91).

As an exercise of body and mind, the mechanism of TCC is complex and evidence from basic research is lacking, thus we roughly discuss it. Resting-state functional magnetic resonance images of TCC experts found that TCC practitioners had greater functional homogeneity in the right post-central gyrus (PosCG), and less functional homogeneity in the left anterior cingulate cortex (ACC) and the right dorsal lateral prefrontal cortex (DLPFC) (92). The PosCG affects pain that its partial resection reliefs of severe limb pain (93). The posterior cingulate cortex is activated by visuospatial imagery (94). The caudal ACC is associated with the complex social interactions (95). The left ACC regulates the hypothalamic-pituitary-adrenal (HPA) axis (96). The prefrontal cortex is particularly important for cognitive control, and the DLPFC may reflect the expression of task goals (97, 98). In breast cancer patients, TCC may establish visuospatial imagery of the movements to posterior cingulate cortex, set task goals through the DLPFC, and reduce pain through the ACC. TCC may also mediate the HPA axis (99). The HPA axis plays a key role in suppressing and shaping immune responses (100, 101). Dysregulation of the HPA axis and the increased levels of pro-inflammatory cytokines [interleukin (IL)-6 and tumor necrosis factor (TNF-α)] could produce fatigue (102). TCC decreases cortisol, IL-6 and TNF-α in cancer survivors that might reduce cancer-related fatigue (35, 37, 103). TCC increases oxygen intake that might improve shoulder function and strength of in patients with breast cancer (104).

Limitations were also in this systematic review and meta-analyses. First, TCC interventions had Yang-style, Chen-style, simplified 24-action and some movements of TCC with different session length, weekly frequency and duration. The eligible studies had clinical heterogeneity because of different TCC interventions. Second, treatments such as chemotherapy, radiation therapy, hormone therapy, and surgery for breast cancer unlimited in this review might restrict the activity of patients to complete TCC. Third, the small quantity and low quality of eligible studies in each meta-analysis might produce risk of bias, inconsistency, imprecision, and publication bias. Finally, although main English, Chinese, Japanese, Korean, and Thai databases were searched, some published or gray literatures might have been missed and all the meta-analyses included <10 studies that publication bias might exist.

## Conclusion

In this systematic review and meta-analysis, TCC had positive effects on QOL, pain, shoulder function, strength of arm, anxiety, and fatigue in breast cancer patients compared with the non-exercise therapy. TCC may have an improvement on QOL, physical function and psychological health in breast cancer patients. Due to the risk of bias in each study, further additional randomized controlled trials with a rigorous methodology and low risk of bias are needed to provide more reliable evidence.

## Author Contributions

YT designed the study, interpreted data, and wrote the manuscript. X-CL, JL, JF, and H-YY contributed to literature search, figures, data collection, data analysis, data interpretation, and draft manuscript. LS, M-LL, LL, JY, X-LQ, and C-ZT contributed to data analysis and data interpretation.

## Conflict of Interest

The authors declare that the research was conducted in the absence of any commercial or financial relationships that could be construed as a potential conflict of interest.

## Abbreviations

CAM: Complementary and alternative medicine
CON: Control
FACIT-F, Functional Assessment of Chronic Illness Therapy-Fatigue, FACT-B: Functional Assessment of Cancer Therapy–Breast
GRADE, Grades of Recommendations Assessment: Development and Evaluation
MOS SF-36: Medical Outcomes Study 36-Item Short-Form Health Survey
QOL: Quality of life
RRT: Routine rehabilitation training
TCC: Tai Chi Chuan
WHOQOL-BREF: World Health Organization Quality of Life Brief Questionnaire.
