# Supplementary material for: Effect of Tai Chi Chuan in Breast Cancer Patients: A Systematic Review and Meta-Analysis
Source: Front Oncol. 2020 Apr 23;10:607. doi: 10.3389/fonc.2020.00607 (PMC7191057; doi:10.3389/fonc.2020.00607)
Supplement: Supplementary file 2 [file Table_2.docx]

**Supplement Table 2. Search strategy in Pubmed-MEDLINE**

| Number | Strategy |
| --- | --- |
| #1 | "tai ji"[MH] |
| #2 | "tai*chi*"[ALL] OR "tai*ji*"[ALL] |
| #3 | #1 OR #2 |
| #4 | "breast neoplasms"[MH] |
| #5 | "breast tumor"[ALL] OR "breast neoplasm"[ALL] OR "breast cancer"[ALL] OR "breast carcinoma"[ALL] |
| #6 | #4 OR #5 |
| #7 | #3 AND #6 |
| #8 | "randomized controlled trial"[PT] |
| #9 | "controlled clinical trial"[PT] |
| #10 | randomized[TIAB] |
| #11 | placebo[TIAB] |
| #12 | randomly[TIAB] |
| #13 | trial[TIAB] |
| #14 | groups[TIAB] |
| #15 | #8 OR #9 OR #10 OR #11 OR #12 OR #13 OR #14 |
| #16 | #7 AND #15 |
